# Synergistic Enhancement of the Percolation Threshold in Hybrid Polymeric Nanocomposites Based on Carbon Nanotubes and Graphite Nanoplatelets

**DOI:** 10.1186/s11671-017-1909-z

**Published:** 2017-02-21

**Authors:** I. Sagalianov, L. Vovchenko, L. Matzui, O. Lazarenko

**Affiliations:** Department of Physics, Taras Shevchenko National University of Kyiv, Volodymyrska Str., 64/13, Kyiv, 01601 Ukraine

**Keywords:** Percolation, Monte Carlo, Nanotubes, Nanoplatelets, Synergistic effect

## Abstract

Synergistic effect causes significant decreasing of the percolation threshold in the ternary polymer composites filled with carbon nanotubes (CNT) and graphite nanoplatelets (GNP) in comparison with binary ones. Enhancement of the percolation threshold strongly depends only on the relative aspect ratios of the filler particles due to the formation of the bridges between puddles of the different filler components. Conditions of both appearance and fading away of the synergistic effect are investigated depending on the relative morphology of CNT or GNP components of the filler. Different lateral sizes, aspect ratios, and volume concentrations of both CNT and GNP in the selected ternary composites were considered. Conditions of the effective substitution of GNP with CNT and vice versa in equal volume concentrations without enlarging of the percolation threshold were established. The results are obtained numerically using the Monte Carlo simulation of the percolation threshold of the different ternary composites.

## Background

Different types of the carbon nanoparticles are well known and promising candidates as filler in polymer composites. Extraordinarily, electrical properties and specific shape with high aspect ratio (AR), relatively small volume concentrations of carbon nanotubes (CNT) or graphite nanoplatelets (GNP) required for percolation threshold achievement, allow us to produce composites with excellent percolation threshold, responsible for mechanical, thermal, and electrical properties of a composite [1–8].

Hybrid ternary composites consisted simultaneously of both CNT and GNP with polymer are able to improve performance and reduce cost of polymer matrix composites and thus are increasingly used as multifunctional materials [5, 6, 9–14]. However, the existing experimental data give us contradictory information about the influence of the parameters of CNT and GNP like relative ARs, diameter *d*_GNP_, length *l*_CNT_, and volume parts of each filler component on the various properties of the ternary composites. The combination of CNT and GNP in the ratio 8:2 (*l*_CNT_ ≈ 10μm, AR_CNT_ ≈ 700, *d*_GNP_ = 4~5μm, and AR_GNP_ = 4000~5000) causes synergistic enhancement of various physical properties and reduces the electrical percolation threshold of the epoxy composites. The main source of this enhancement treated as ease formation of a conductive network due to the improved state of CNT dispersion in the presence of GNP [5]. Thermal conductivity can also be significantly enhanced in the ternary composites with 3:1 GNP vs CNT ratio in wt. % compared to binary ones (*l*_CNT_ = 300~1000 nm, AR_CNT_ = 180~300, *d*_GNP_ = 200~1000 nm, and AR_GNP_ = 50~300) [6]. On the other hand, the influence of the exact morphology of CNT and GNP on synergistic effect was not fully investigated. Ratios between each of the compounds in ternary composites are fragile and easily affected by exact morphology of the GNP or CNT. Quite likely that particles with another morphology parameters like AR can significantly change these CNT:GNP “golden ratios” (8:2 [5], 3:1 [6], or even 1:1 [10–12]) and, hence, eliminate all synergistic enhancement of the percolation threshold of a ternary composites.

A three-dimensional (3D) Monte Carlo model [15–17] was implemented in C++ and Java environments for predicting of the percolation threshold of polymer composites filled with conductive graphite nanoplatelets and/or carbon nanotubes. The conductive fillers are modeled as a 3D network of finite sites that are randomly positioned. The percolation behavior of the network was studied using the Monte Carlo method, which leads to the determination of a critical filler volume fraction (percolation threshold *φ*_*с*_). Several controlling parameters for different types of filler, namely, the nanotubes (nanoplatelets), length (diameter), and their aspect ratio are taking into account. Since the production of nanotubes is more expensive than the production of disk-like graphite nanoplatelets, the aim of our study is also the investigation of the possibility of effective substitution of the nanotubes with nanoplatelets (or vice versa) taking into account losses in percolation threshold.

The dependence of the percolation threshold on the morphology of the filler components, as well as the effect of the simultaneous presence of the nanoparticles with a different aspect ratio, were investigated as well as conditions of realizations of the synergistic effect in ternary composites.

### Methods

Connection probability can be represented as probability of pathway establishing in a polymer composite modeled in a 3D cubic representative volume element (RVE) within the Monte Carlo approach [15–18]. The main difficulty of this methodology is obtaining of an intersection between different particles of the filler.

#### Monte Carlo Approach for Calculation of the Percolation Threshold: Algorithm Consruction

Calculation of percolation threshold of composites filled with different setups of nanotubes or/and nanoplatelets by utilizing of the simple algorithm consisted of several steps:

1. Generation of a filler distribution for selected volume fractions of CNT and/or disks.
2. Evaluation of an intersection between each two particles of the filler by utilizing of the Perram’s contact function [17, 19].
3. Checking of the intersection data in order to find established pathway between two opposite sides of the RVE.
4. Repeat steps 1–3 from 100 to 1000 times depending on selected simulation conditions and parameters for different spatial distribution of a filler in order to get an averaged connection possibility (percolation threshold corresponds to the 50% probability of the connection).

The most time-consuming procedure is determination of overlapping between different filler particles with computational efforts scaled with number of particles in the system as N!.

#### Contact Function for Two Nonequivalent Ellipsoids

We neglected the tunnel effect because of low filler volume fraction. Hence, during our calculations, we utilized a well-known model of softcore filler particles able to penetrate each other. In order to minimize computational efforts, we modeled nanoplatelets (nanotubes) as oblate (prolate) spheroids with different length of semiaxes for selected aspect ratios. Intersection of each two particles of the filler was obtained with Perram’s contact function [17, 19].

1 $$ {F}_{A B}=\lambda \left(1-\lambda \right){r}_A^T C\left(\lambda \right){r}_B, $$

where *r*_**A**,**B**_ are vectors defined centers of two ellipsoids (A or B) in RVE. The parameter *λ* is restricted to the interval 0 < λ < 1, so that we have *F*_*AB*_ ≥ 0. By variation of the *λ*, we determine the maximum of the function *F*_*AB*_ [19]. This maximal value indicates intersection between two ellipsoids with the following condition:

2 $$ \underset{0<\lambda <1}{ \max }{F}_{AB}\left\{\begin{array}{c}\hfill <1,\kern0.75em \mathrm{overlapping}\kern1.75em \hfill \\ {}\hfill =1,\kern0.75em \mathrm{tangent}\kern3.5em \hfill \\ {}\hfill >1,\kern0.75em \mathrm{non}\ \mathrm{overlapping}\hfill \end{array}\right. $$

By utilizing *R*_*i*_(*Ω*_*A*,*B*_) (vectors define space orientation and length of semiaxes of *A* and *B* ellipsoids), we must obtain matrix *C*(*λ*) with the following relations [19]:

3 $$ C\left(\lambda \right)={\left[\lambda A+\left(1-\lambda \right) B\right]}^{-1} $$

4 $$ A\left({\varOmega}_A\right)={\displaystyle {\sum}_{i=1}^3{R}_i\left({\varOmega}_A\right){R}_i^T\left({\varOmega}_A\right)} $$

5 $$ B\left({\varOmega}_B\right)={\displaystyle {\sum}_{i=1}^3{R}_i\left({\varOmega}_B\right){R}_i^T\left({\varOmega}_B\right)} $$

This contact function is suitable for intersection analysis of unequal ellipsoids (contrary to Vieillard–Baron’s contact function made for identical ellipsoids [20]) and provides one of the fastest ways of the Monte Carlo simulations of the percolation threshold.

#### Dependence of Simulation Results on the Size of Filler Particles and RVE

**Fig. 1:**
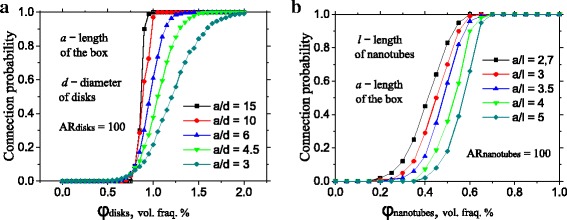
Dependence of the connection probability on the filler volume for different *a*/*l*_GNP_ or *a*/*l*_CNT_ ratios for nanoplatelets (**a**) or nanotubes (**b**) acting as a separate filler

Varying of the filler particle size compared to the RVE leads to the anticipated change of the percolation threshold [15]. The dependence of the percolation threshold on the filler volume for different RVE side (*a*) vs disks diameter (*d*_GNP_) ratio; *a*/*d*_GNP_ is depicted on Fig. 1a. Percolation threshold corresponds to the 50% connection probability and slightly decreases with *a*/*d*_GNP_ ratio growth. Saturation point is 0.6 vol. % of the nanoplatelets with aspect ratio of 100. In most part of the calculations, we used different ratios (*a*/*d*_GNP_ = 5~25) in order to decrease computational efforts. Small size of the filler particles causes the significant enlargement of their amount required to obtain connection between the opposite sides of the RVE.

Same reasons were considered in choosing of the aspect ratio of the nanotubes. We investigate nanotubes with unusually small (AR = 50) aspect ratio in the biggest part of calculations. Size of these nanotubes is bigger than size of the ones with higher AR—hence, smaller amount of nanotubes is required for a percolation achievement. On the other hand, nanotubes with huge AR (700~1000) tend to merge into puddles (agglomerations) with enlarging of the percolation threshold value which partially compensates its extraordinary morphology.

Contrary to this tendency, modeled nanotubes are distributed in not viscous, frictionless space. These approximations drastically enhance (decrease) percolation threshold. In order to compensate agglomeration and viscosity, we modeled nanotubes (and puddles of the nanotubes) with effective spheroids with small (50–200) aspect ratios.

As we showed on Fig. 1b, dependence of the connection probability for nanotubes with AR = 100 on the *a*/*l*_CNT_ ratio exhibits slow saturation up to the 0.7% in vol. %. However, recommended values of *a*/*l*_CNT_ during simulations are significantly smaller than ones for spheroids [15]. Simulations of the connection probability for systems with *a*/*l*_CNT_ > 5 and AR > 200 consume extremely huge time; hence, we utilize *a*/*l*_CNT_ = 5. Obtained percolation threshold for nanotubes equals 0.56% (Fig. 1b) and coincides with other numerical simulations [15].

Comparison of simulation results with experimental values for the percolation threshold of the composites filled with nanoplatelets. Different AR of nanoplatelets were considered.

As we can conclude from Table 1 (experimental results of the critical volume of composite consisted from the epoxy filled with the graphite nanoplatelets [21–26]), obtained simulation results for nanoplatelets (modeled as oblate spheroids) are slightly smaller than experimental ones. This can be easily explained with two factors. First factor is viscosity of the polymer. Simulation results correspond to the filler particles distributed in a free space without viscosity. On the other hand, the more viscous polymer will be used—the bigger amount of filler is required for percolation threshold achievement. Second factor is difference between modeled spheroids and realistic form of the nanoplatelets. Obviously, nanoplatelets have much more complicated form than simple disks (spheroids). The difference between experiment and simulation grew with the aspect ratio of nanoplatelets. This tendency witnesses the enlargement of the difference between the shape of spheroids and realistic nanoplatelets with aspect ratio growth. Hence, in most of simulations, we utilized relatively small aspect ratios of the nanoplatelets (up to 200). However, despite these significant approximations, our results exhibit good quality agreement with available experiment (Table 1) for different aspect ratios of GNP.

**Table 1:** Comparison of simulations with experiment

AR of the nanoplatelets, vol. %	10	20	50	100	200
Percolation threshold, experiment	9.5	5.0	3.0	1.4	0.8
Percolation threshold, simulation	8.7	4.2	2.0	0.9	0.5

## Results and Discussion

In order to fully cover all sides of the simultaneous influence of both nanotubes and nanoplatelets on the percolation threshold, we divided our simulations into several parts. Each of these parts corresponds to the one factor of tuning the physical properties of the polymer composites with a mixed nanotube/nanoplatelet filler. Most of these results obtained for the modeled filler particles like nanotubes with relatively small AR = 50. However, this approximation is reasonable in terms of qualitative analysis of the percolation threshold in ternary polymer composites. On the other hand, our nanotubes with small AR are ideally distributed in nonviscous space without interweaving or tangling contrary to the realistic CNT in polymer. All these facts partially compensate the difference between realistic CNT and our modeled “effective” CNT with small AR acting as agglomerations of realistic ones.

### Percolation Threshold for Composites with Different ***l***_CNT_/***d***_GNP_ Ratio

**Fig. 2:**
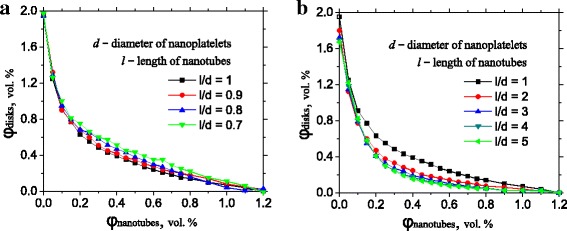
Dependence of the percolation threshold on the relative size of nanoplatelets and nanotubes in ternary composites. **a** The length of the nanotubes is smaller than the diameter of nanoplatelets. **b** The length of the nanotubes is bigger than the diameter of nanoplatelets

Sizes of the different particles in the ternary filler are usually similar [5, 6] and can be controlled by the conditions of synthesis. The dependence of percolation threshold on *l*_CNT_/*d*_GNP_ ratio is complicated. In order to investigate the influence of relative *l*_CNT_/*d*_GNP_ ratio on percolation threshold, we considered modeled ternary composite with AR_CNT_ = AR_GNP_ = 50. As we can see from Fig. 2b, ratio *l*_CNT_/*d*_GNP_ > 1 increases synergistic effect and enhances percolation threshold. This enhancement slowly saturates with *l*_CNT_/*d*_GNP_ = 5. On the other hand, ratio *l*_CNT_/*d*_GNP_ < 1 causes enlarging of the percolation threshold compared to *l*_CNT_/*d*_GNP_ = 1 (Fig. 2a). The source of these modifications can be the same as on Fig. 1. Different values of *a*/*l*_CNT_ and *a*/*d*_GNP_ give slightly different values of the connection probability. This can be easily noticed on Fig. 2b for 0.0 vol. % of nanotubes. Value of the percolation threshold decreases from 1.9 to 1.7 vol. % while diameter of the GNP decreases in five times. Variation of the ratio between diameter of the GNP (length of the CNT) and size of the RVE is able to change connection probability and hence percolation threshold accordingly to Fig. 1. As a consequence, we can state that variation of *l*_CNT_/*d*_GNP_ causes almost no influence on the percolation threshold of the ternary polymer composite.

### Percolation Threshold for Composites with Different Aspect Ratios of Nanotubes or Nanoplatelets

**Fig. 3:**
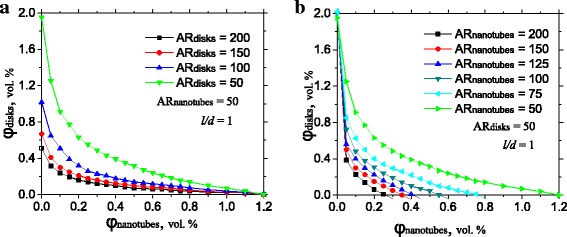
Dependence of the percolation threshold on the aspect ratio of nanoplatelets (**a**) and nanotubes (**b**) in modeled ternary polymer composite

High aspect ratios of a filler particles provide better values of the percolation threshold. However, fabrication of the filler consisted with identical nanotubes (nanoplatelets) is difficult and expensive. Usually, filler contains particles with specific range of a possible aspect ratios and lateral sizes. Percolation thresholds of the mixed filler with fixed AR_GNP_ of graphite nanoplatelets and different AR_CNT_ of nanotubes (and vice versa) are depicted on Fig. 3. In order to investigate the influence of AR variation on the percolation threshold, we considered filler with *l*_CNT_/*d*_GNP_ = 1 in all simulations. With enlargement of the AR of each type of particles in mixed filler, percolation threshold became smaller (Fig. 3), which witnesses about anticipated enhancement of the filler quality. Percolation threshold of the ternary composite can be effectively decreased proportionally to the AR_CNT_/AR_GNP_ ratio. AR_CNT_/AR_GNP_ = 2 gives twice smaller percolation threshold; AR_CNT_/AR_GNP_ = 4 or AR_GNP_/AR_CNT_ = 4 gives four times smaller percolation threshold, etc. This tendency remains almost unchanged both for nanoplatelets (Fig. 3a) or nanotubes (Fig. 3b) with high AR.

### Conditions of Synergistic Effect in Ternary CNT/GNP/Polymer Composites

**Fig. 4:**
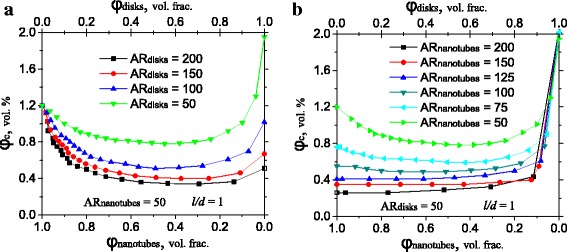
Conditions of the synergistic effect in mixed nanotubes/nanoplatelets filler. Percolation threshold versus volume fraction of the CNT or GNP. Different AR of nanoplatelets (**a**) or nanotubes (**b**) are considered. Each point corresponds to the unique composite with its own aspect ratios of CNT or GNP and volume part of each filler compound

Synergistic effect plays important role in fabrication of ternary composites. Different experiments state that synergism between different compounds of the filler occurred only with selected volume or weight ratios between them, like 8:2 [5], 3:1 [6], or 1:1 [10–12]. In order to establish optimal conditions for realization of this effect, we studied ternary composite with static *l*_CNT_/*d*_GNP_ = 1, AR_GNP_ = 50, and different values of aspect ratios of the nanotubes in range AR_CNT_ = 50~200. Percolation threshold (*φ*_с_) in binary GNP/polymer composite (Fig. 4b, left side) is the same for all curves because of the absence of CNT: *φ*_с_ ≈ 1.95 vol. %. On the other hand, in binary CNT/polymer composite (Fig. 4, right side), *φ*_с_ ≈ 0.24~1.2 dependent on the AR_CNT_. Values of the *φ*_с_ for ternary CNT/GNP/polymer composite are lesser than those of the *φ*_с_ in binary CNT/polymer or GNP/polymer because of appearing of the synergistic effect. Now, let us consider more precisely several different modeled composites.

As we can see from Fig. 4b, ternary composite with AR_CNT_/AR_GNP_ = 50 (highest curve, green color) has worst *φ*_с_ because of low AR of both compounds of the fillers. On the other hand, even minor volume fraction of nanotubes or nanoplatelets in ternary CNT/GNP/polymer composite allows to decrease percolation threshold compare to the respective CNT-based (*φ*_с_ ≈ 1.2 vol. %) or GNP-based (*φ*_с_ ≈ 1.95 vol. %) binary composites. Calculated percolation threshold values (in vol. %) for several different CNT:GNP ratios are quite comparable with each other: *φ*_с_ ≈ 1.0 for 1:9, *φ*_с_ ≈ 0.9 for 2:8, *φ*_с_ ≈ 0.8 for 4:6, *φ*_с_ ≈ 0.9 for 8:2, etc. As we can conclude, synergistic effect allows us to decrease percolation threshold approximately on 35% compared to CNT and on 60% compared to GNP-based binary composites in a wide range of CNT:GNP vol. frac. ratios.

However, this is valid only for the ternary composite with similar morphology of the CNT and GNP. Now, let us consider filler particles with different AR. Some restrictions appear at huge difference between AR of filler compounds. Enhancement of the percolation threshold due to the synergistic effect slowly degrades with increasing of the difference between AR_CNT_ and AR_GNP_. Synergistic enhancement gets its minimal value for AR_CNT_ = 100 and AR_GNP_ = 50 (aspect ratio of the nanotubes twice bigger then aspect ratio of the graphite nanoplatelets) and completely disappears if AR_CNT_ = 200 and AR_GNP_ = 50 (Fig. 4b). Nevertheless, small concentration of CNT (0.2 in vol. frac.) allows to decrease percolation threshold in five times compared to binary GNP/polymer composite and to achieve almost the same *φ*_с_ as in the 100% CNT/polymer composite. Since CNT are way more expensive than GNP, substituting of 80% of CNT on GNP (with small AR) will drastically reduce the cost of the polymer composite.

The same results were obtained for a ternary composite with fixed AR_CNT_ = 50 and different AR_GNP_ = 50 ~ 200 (Fig. 4a). Synergistic effect reaches its maximum value in case of similar aspect ratios (AR_GNP_ = AR_CNT_ = 50) and drastically decreases with increase of the difference between aspect ratios of filler components. With enlarging of the AR_GNP_, percolation threshold behaves exactly like in previous case (Fig. 4b). Decrease of the synergistic effect on Fig. 4a is slower compared to the results on Fig. 4b due to the difference in the morphology of the nanoplatelets and nanotubes.

Summing up, let us consider ternary composite with high AR_GNP_ and small AR_CNT_—for example, multilayer graphene flakes and cumbersome nanotubes with low aspect ratio. We still can effectively substitute some of the graphene flakes with nanotubes and without significant losses in *φ*_с_, but no synergism will be observed.

These results indicate that the presence of the CNT serves as additional tool of bounding between different GNP puddles and vice versa. From this point of view, if one of the filler components has smaller AR than another one, percolation threshold of the mixed filler will significantly degrade. Only particles with similar aspect ratios are capable to mutually complement each other. Also, it is important to notice that existing experimental explanation of synergism affects other aspects of percolation in ternary composites. Results seem to indicate that GNP could prevent re-aggregation of the CNT bundles [5] and hence increase electroconductivity. Synergism originates from the bridging of planar nanoplatelets by the flexible CNT which leads to a decreased thermal interface resistance along the (2D-1D) hybrid filler network due to the extended area of the SWNT-GNP junctions [6]. Our results witness that the main condition of appearing of strong synergism between different components of the filler is similarity of their aspect ratios. The bigger difference between ARs we have, the smaller synergism can be observed. Other morphology parameters of CNT or GNP are insignificant.

## Conclusions

By employing numerical calculations within the Monte Carlo approach, we systematically studied the percolation threshold in different ternary CNT/GNP/polymer composites. We conclude as follows.

(i) Synergistic effect allows us to significantly decrease percolation threshold in ternary composites compared to binary CNT- or GNP-based ones. Enhancement of the percolation threshold strongly depends on the relative AR of the filler particles due to the formation of the bridges between different filler component puddles. Synergism reaches its maximum value in case when AR_CNT_ = AR_GNP_ and completely disappears if AR_CNT_ > 2AR_GNP_ or vice versa.
(ii) Relative ratio of the sizes of the filler particles *l*_CNT_/*d*_GNP_ has almost no influence on the percolation threshold of the ternary polymer composites and is insignificant for the realization of the synergistic effect.
(iii)   Percolation threshold of the ternary composites can be effectively decreased proportionally to the AR_CNT_/AR_GNP_ if AR_CNT_ > AR_GNP_ or vice versa with linear behavior.
(iv)   Particles of one filler component can mutually be substituted by particles of the other filler component with the same or close aspect ratio without significant losses in percolation threshold (even after substitution up to the 80% of CNT on GNP with smaller AR).

## Abbreviations

3D: Three-dimensional
*a*: Length of the representative cubic volume element sides
AR: Aspect ratio
AR_CNT_: Aspect ratio of the carbon nanotubes
AR_GNP_: Aspect ratio of the graphite nanoplatelets
CNT: Carbon nanotubes
*d*_GNP_: Diameter of the graphite nanoplatelets
GNP: Graphite nanoplatelets
*l*_CNT_: Length of the carbon nanotubes
RVE: Representative volume element
*φ*_c_: Percolation threshold (critical volume of the filler)
